# Only a topological method can identify *all possible* network structures capable of Robust Perfect Adaptation

**DOI:** 10.1371/journal.pcbi.1011638

**Published:** 2023-11-22

**Authors:** Robyn P. Araujo, Lance A. Liotta

**Affiliations:** 1 School of Mathematical Sciences, Queensland University of Technology, Brisbane, Queensland, Australia; 2 Center for Applied Proteomics and Molecular Medicine, George Mason University, Manassas, Virginia, United States of America; Pázmány Péter Catholic University: Pazmany Peter Katolikus Egyetem, HUNGARY

Robust Perfect Adaptation (RPA), or robust homeostasis, is a ubiquitous property of biological systems, and ensures that key properties of the system can reset themselves in response to environmental disturbances, and maintain a fixed value (the system’s ‘setpoint’) at steady-state. RPA is a structural property of biological networks, and is independent of special parameter choices–hence, ‘robust’. Simple network configurations that can support RPA have been known for several decades [1–3], but until recently all known RPA-permissive network designs were limited in size and complexity to fewer than five interacting molecules. By contrast, almost all molecular networks of functional importance to living organisms comprise far more than five molecules, particularly in the context of cellular signal transduction and cellular metabolism, where hundreds or even thousands of distinct proteins or phospho-forms can dynamically assemble into vast and intricate networks of molecular interactions. How could the extraordinarily complex networks that have self-organized and diversified throughout evolution be compatible with such robust and sophisticated functions as RPA?

Over the past several years, we have provided definitive answers to this question [4–6]–both at level of the network macroscale [4], in terms of a complete and exhaustive set of network design principles or ‘topologies’ that are compatible with RPA, and in terms of the intricate intermolecular interactions that make the requisite ‘computations’ at the microscale of biochemical reaction networks [7]. As a consequence of these discoveries, RPA now stands alone as a fundamental biological response for which there exists a universal explanatory framework at the molecular level and an exhaustive set of network design principles^7^, encompassing single-input/single output-networks subjected to constant-in-time disturbances, under the assumption of stability.

*PLOS Computational Biology* recently published an article by Bhattacharya et al. [8] which claims to ‘discover’ RPA-capable biological network structures using control theoretic approaches. Since we ourselves had previously published a complete solution space for all possible RPA-capable network architectures, we would like to provide some additional clarification on this new article [8] in the context of our more general and comprehensive prior study [4]. We also seek to provide an expanded view of the many exciting developments in this field, and the grand challenges for the future.

**Fig 1:**
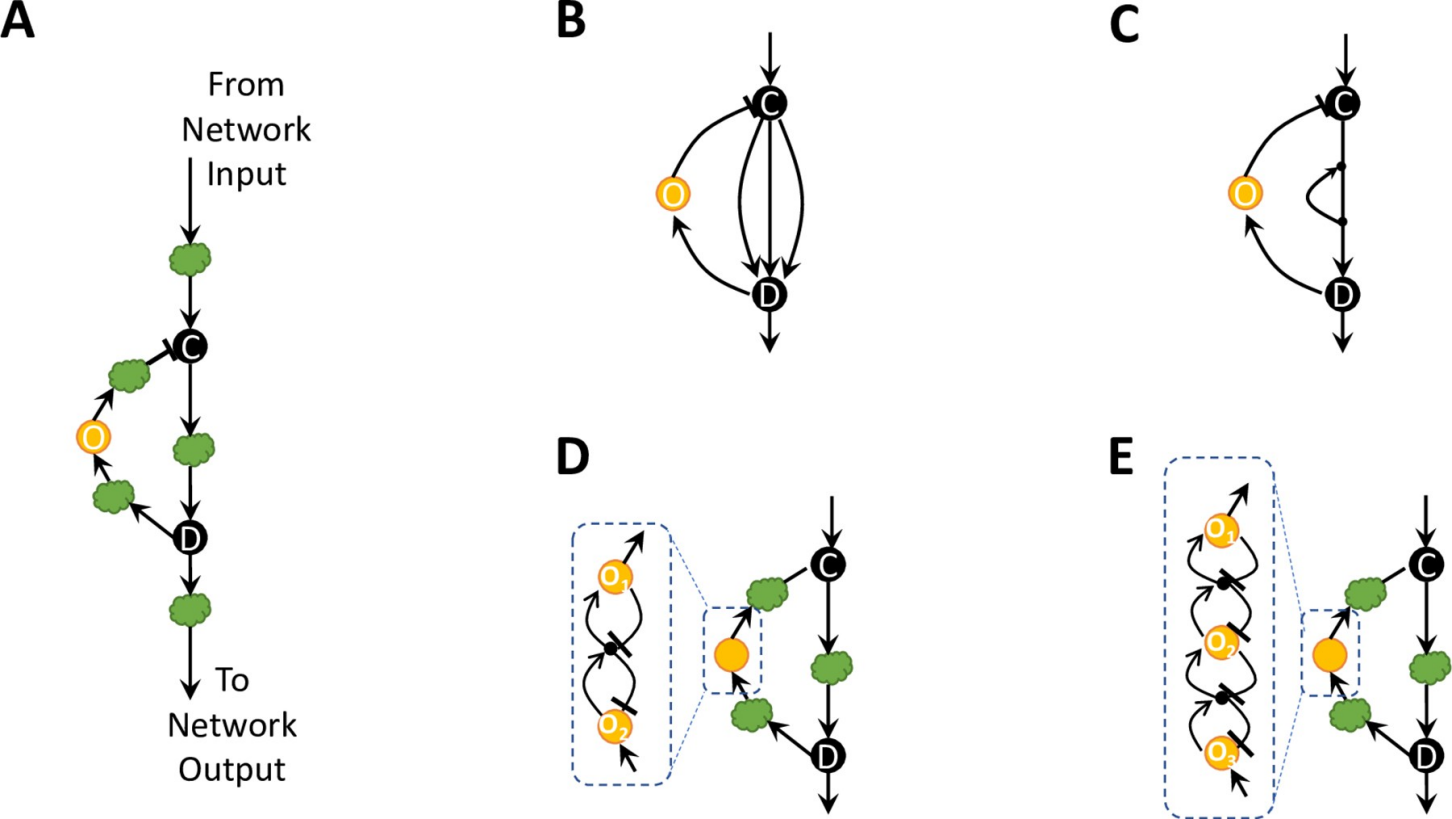
Fundamental Design Principles of Opposer Modules. **A**. General structure of an Opposer module, incorporating the simplest case of a single opposer node, O, for illustrative purposes. The opposer node (yellow) must be endowed with special mathematical properties, known as ‘opposer kinetics’. Arbitrary network regulations or subnetworks can be incorporated or embedded at the locations denoted by the green clouds. Node C, the ‘connector node’, and node D, the ‘diverter’ node distinguish the ‘route’ segment(s) from the ‘feedback’ segment of the module’s overarching feedback structure. **B.** An Opposer module with single opposer node (O), for which the embedded regulations between nodes C and D consist of three parallel routes. The module thereby consists of three feedback loops with a shared feedback segment, each with a unique route segment. As noted, the requisite opposer node must be embedded into the feedback segment of the loop. **C.** An Opposer module with single opposer node (O), for which the embedded regulations between C and D consist of a single feedback loop. Note that an opposer node cannot be embedded into this feedback loop, since the ‘outer’ feedback portion delineated by nodes C and D already contains an opposer node. Multiple opposer nodes can only be incorporated into a single Opposer module via the special structure of an opposing set. **D.** An Opposer module with a two-node opposing set embedded into the feedback segment of the module. **E**. An Opposer module with a three-node opposing set embedded into the feedback segment of the module.

First and foremost, we acknowledge that the Bhattacharya et al. article [8] develops a systematic control-theoretic approach, along with an analysis of stability, which is able to identify a variety of RPA-capable network structures in addition to requirements for non-zero sensitivity in these networks. The authors are also able to show that RPA minimizes the time taken for a network to attain its peak response. But the network structures identified as RPA-promoting in the Bhattacharya et al. article [8] are all encompassed by our earlier study [4], which presents a general topological method for identifying the full solution space of all possible RPA-capable network structures. We wish to clarify for the readership of *PLOS Computational Biology* that all RPA-capable networks–regardless of size or complexity–are necessarily modular in nature. Large and highly complex RPA-capable networks may be decomposable into many such ‘basis modules’, which can admit rich and complex realizations, but which can only be drawn from two distinct and well-characterized classes: *Opposer modules* and *Balancer modules*. Particularly simple RPA-capable networks, such as the three-node networks discovered by Ma et al. [3] by computational screening, can consist of a single such module. Unfortunately, Bhattacharya et al. [8] misrepresent and mischaracterize these fundamental RPA basis modules throughout their paper, and consistently refer to Balancer modules when describing the properties of Opposer modules, and vice versa, and also fail to recognize the full topological complexity of these fundamental network building blocks. Contrary to the claims of the Bhattacharya et al. [8] article when referring to our prior study [4] we by no means “conjectured that a balancer module should contain at least one negative feedback …” for stability reasons or otherwise. Balancer modules do not, in fact, require a negative feedback loop for stability, or to achieve RPA. To clarify this important distinction, we refer interested readers to the comprehensive and highly-accessible descriptions of the two RPA basis modules in two recent review articles [5, 9]. Below we provide a brief summary of the general properties of Opposer modules–the ones which do strictly require feedback for their RPA-conferring functions (see Fig 1).

The distinguishing feature of all Opposer modules is the existence of at least one special computational node whose ‘kinetic multiplier’ [4] (a real-valued function associated to each node in a network, which encodes important mathematical properties of the reaction kinetics at the node [4]) vanishes everywhere on the steady-state locus of the system. Computational nodes with this special property are referred to as *opposer nodes*; all other nodes in a network are characterized by non-zero kinetic multipliers. In our paper [4], we prove that an opposer node can only exist within a feedback loop (Theorem 1 in [4]). We also prove that an opposer node must be located within the feedback segment of the feedback loop, not the route segment, in order to contribute its RPA-promoting functions to the network (Theorem 2 in [4]). We develop a generalization of this idea to *opposing sets* (Theorem 3 in [4]), accounting for the possibility that Opposer modules can contain multiple independent opposer nodes. Note that a single Opposer module can only contain multiple opposer nodes if these are organized into an opposing set (see Fig 1)–a special embedded architecture involving interlinked feedback loops, each containing exactly one opposer node. Opposer modules can certainly contain additional feedback loops (*without* an opposer node) embedded into the route segments of the module (delineated by a connector node at its apex, and a diverter node at its base, as depicted in Fig 1), for example, or embedded into the feedback segments on either side of the opposer node (D➔O, or O➔C). These additional (optional) feedback loops are entirely compatible with RPA (on the assumption of stability), but are not required for RPA capacity.

Contrary to the claims of the Bhattacharya et al. [8] paper, the kinetic multiplier associated to an opposer node does not generally achieve a zero value on the steady-state locus through special parameter conditions. Rather, the computational properties of opposer nodes–and indeed balancer and connector nodes, which accomplish the RPA-promoting computations of Balancer modules–are independent of specific parameter choices, and are conferred by the special structural properties of the underlying chemical reactions. The antithetic integral controller [2, 10] provides a particularly striking illustration of this concept, and more recently, universal structural conditions for computational nodes in RPA networks have been discovered [7, 11]. The computational study by Ma et al. [3], which employed the simplification of Michaelian kinetics, considered only ‘almost perfect’ adaptation. Within this simplified biochemical description, the creation of an opposer node corresponds to the vanishing of all Michaelis constants. Thus, many parameter choices are compatible with an adaptive response within the specified tolerance, with smaller Michaelis constants producing smaller steady-state deviations from the output node’s setpoint. But the fact that a vanishing parameter group can mimic the properties of an opposer node is only possible because of the structural properties of the chemical reactions of a covalent modification cycle, with or without positive autoregulation [12, 13], a fact that becomes vividly clear from the discovery of the complete solution space to RPA-permissive chemical reaction structures [7].

Regarding the ‘sign’ of feedback loops within an Opposer module, Bhattacharya et al. [8] are right to suggest that if only one such feedback loop exists, it must be a negative feedback loop—comprising an odd number of inhibitory interactions—in order to promote stability. Our prior paper [4] makes clear that if an Opposer module contains only one feedback loop, then it must contain an opposer node in its feedback segment–precluding the possibility of a direct link between the output (or at least the diverter node of the module) and the input (or connector node); cf. Theorem 4 in [8]. But more importantly, the authors overlook the tremendous additional network complexity that could exist within Opposer modules. We suggest that Bhattacharya et al. [8] could have made a much stronger statement on the requisite signs of feedback loops in RPA-promoting modules: Any feedback loop containing an opposer node *must* be a *negative* feedback loop. This more precise statement encompasses more complex versions of Opposer modules that include opposing sets; it also allows for the possibility that *other* (allowed but inessential) feedback loops (e.g. a small loop embedded into the route segment of an Opposer module–see Fig 1c above) could be positive for certain special parameter regimes, and still produce a stable network. Unfortunately, the method used by Bhattacharya et al. [8] does not allow for the discovery of opposing sets, and is therefore silent on the characteristics of such relatively complex feedback-regulated architectures within Opposer modules.

We clarify for the readers of *PLOS Computational Biology* that Balancer modules never contain opposer nodes, although they can certainly contain feedback loops embedded into their parallel routes. These feedback loops, if they exist, consist exclusively of a special type of computational node known as *balancer nodes*. All Balancer modules also contain a single connector node, which is a third distinguished class of computational node, distinct from both opposer nodes and balancer nodes.

Unlike the Bhattacharya et al. [8] paper, our article [4] places little emphasis on stability, since in a completely general setting–accounting for the full solution space of RPA-capable designs that we identify in our study [4]–stability is an exceedingly complicated matter. Indeed, stochastic implementations of certain RPA-promoting molecular interactions, such as antithetic-integral control [2, 10, 11, 14], can admit stable performance even when the corresponding deterministic model is unstable. Our brief closing remarks on stability in Supplementary Note 8 in [4] underscore the tremendous difficulty of drawing definitive conclusions as to stability for general parameter regimes for large and highly complex RPA-promoting network designs that could feature many feedback loops. These remarks do not constitute a “conjecture”. Assuming that any feedback loop containing an opposer node is negative, then stability also depends on feedback loop parameters and even the magnitude of the disturbance/stimulus to which the network is expected to adapt [15]. In practice, evolution will quickly discard unstable networks, and will instead traverse a network design space that engenders stability for a suitably wide range of parameter regimes and network disturbances that are relevant to the biological context at hand.

The overarching technical difficulty with the Bhattacharya et al. [8] study is that it is unable, by its very nature, to offer a completely general picture of the network design space for RPA. Curiously, the authors claim that we “developed a graph-theoretic method to address (this) problem”. In actual fact, the cornerstone of our method was to *first* interpret the individual signed terms of the RPA equation (the algebraic condition for RPA, comprising an (*n*−1)-th order polynomial of up to (*n*−1)! terms for an *n*-node network) as points in a topological space, together with collections of ‘independently-adapting subsets’. This collection of subsets constitutes a topology on the terms of the RPA equation. We proved that a ‘minimal’ such subset could belong to one of two distinct forms: S-sets and M-sets. Only through a comprehensive analysis of the permissible algebraic structures of these two classes of subsets, and by considering the relationship of the contents of these subsets to structural properties of the associated network, could a topological basis for any RPA equation be definitively established. And only by relating these topological basis sets to their corresponding network architectures (modules), could we arrive at an exhaustive description of all possible RPA-capable network designs. In doing so, we identified novel network features of RPA-promoting modules, such as opposing sets (see ^4–7^ for further details), and also rigorously established the intermodular connectivity rules that allow arbitrarily large networks to achieve RPA through multi-modular network designs.

Interestingly, Bhattacharya et al. [8] claim in their Supporting Information that “there are two ways in which all of the terms of (the RPA Equation) sum up to zero, (1) All the terms are zero individually. (2) There exist terms with equal and opposing actions.” What the authors are actually describing here are the properties of a single ‘independently-adapting subset’ of an RPA Equation, not the RPA Equation as a whole, in complete generality. Indeed, their point (1) corresponds to a description of an S-set (albeit without considering the potentially complex internal structures of such sets, which can give rise to *opposing sets*), while point (2) represents a simplified description of an M-set. In this connection, we clarify for the readers of *PLOS Computational Biology* that the network designs considered by Bhattacharya et al. [8], which they claim to hold for networks of any size are, in fact, simply larger versions of the three-node motifs identified by Ma et al. [3], and ignore more complicated realizations of the basis modules and the complex possibilities of multi-modular networks.

Many results and special cases have been ‘rediscovered’ and presented as ‘new’ by Bhattacharya et al. [8], despite being straightforward corollaries of our prior study [4]. For instance, it is clear that RPA could arise in a two-node network in which one node is the input/output node, and the other node is an opposer node (see, for example, Figure 2 and Supplementary Note 2.4 in our paper [4]). This was by no means an open question at the time of the Bhattacharya et al. [8] study. Theorem 3 by Bhattacharya et al. [8] is covered by our exhaustive analysis of M-sets (see, for example, the Supplementary Notes 3.2.3 and 3.2.4 in our prior work [4]), which also recognizes that the collections of parallel routes in a Balancer module *may* contain embedded feedback loops comprised entirely of balancer nodes (*not* opposer nodes); these feedback loops are compatible with RPA, but not required. Their Theorem 3 [8] also fails to recognize that such feedback loops create the possibility of a Balancer module with only coherent feedforward paths (routes)–see also Ma et al. [3] Fig S12 (Supplemental Data, Section 9 in [3]). Theorem 4 [8] follows from the most basic fact about any Opposer module with a single opposer node: the opposer node must reside in the feedback segment of its feedback loop(s), not the route segment, precluding a direct link from output back to input. Since any feedback loop containing an opposer node must be a negative feedback loop, all parallel routes within the route segment of an Opposer module (connecting the connector node to its downstream diverter node–see Fig 1 above) must be coherent. Theorem 5 [8] follows from the fact that any RPA-capable network must contain at least one RPA basis module–either an Opposer module or a Balancer module.

While we recognize that Bhattacharya et al. [8] identify requirements for non-zero sensitivity in RPA-capable networks, and also show that RPA minimizes the time taken for the network to attain its peak response, these findings fall far short of the “discovery” of “adaptation-capable biological network structures” that their title so boldly claims. Moreover, we clarify for interested readers that many forms of robust homeostasis in nature exhibit a special form of RPA known as absolute concentration robustness (ACR) [7, 16] which requires no such non-zero sensitivity. We also note for the interest of readers the several recent landmark contributions to synthetic biology [17, 18] which recognize the unhelpful role of excessive ‘sensitivity’ in the transient responses of RPA networks, and which develop novel strategies to mitigate these potentially pernicious responses.

As we note in a more recent study [7], the quest to identify a universal set of design principles for the complex, self-organizing networks that can exist in nature, throughout all domains of life, is widely considered a brass-ring grand challenge in the life sciences. We hope that our recent discovery of the universal structural requirements for RPA [4–7], along with the abundance of exciting new work in this field [9–13, 17–22], will inspire the readers of *PLOS Computational Biology* to consider the fundamental network principles governing more complex phenotypes–Turing patterning, for instance, or multistability/switching responses. These grand challenges remain open.
